# Prevalence of bean scab caused by *Elsinoë phaseoli* and challenges associated with bean cultivation in Kenya

**DOI:** 10.1002/pei3.70013

**Published:** 2024-10-15

**Authors:** Y. O. Masheti, J. W. Muthomi, W. M. Muiru, E. E. Arunga, P. Gepts

**Affiliations:** ^1^ Kenya Agricultural and Livestock Research Organization Non‐Ruminant Research Institute Kakamega Kenya; ^2^ Department of Plant Science and Crop Protection University of Nairobi Nairobi Kenya; ^3^ Department of Agricultural Resource Management University of Embu Embu Kenya; ^4^ Department of Plant Sciences, Section of Crop and Ecosystem Sciences University of California Davis California USA

**Keywords:** agricultural practices, bean scab management, farmers' knowledge, pest prevalence, smallholder farmers

## Abstract

This study investigated the prevalence of scab caused by *Elsinoë phaseoli* causing yield losses on beans in Kenya. The research focused on common practices and challenges faced by subsistence farmers with the aim of providing insights into scab prevalence, impact, and potential management challenges. A structured questionnaire was employed in a survey conducted in 2022 and 2023, covering major bean‐growing regions using a three‐stage sampling design. Data from 128 bean farmers included information on farm size, seed sources, cropping systems, awareness of challenges, and pest/disease management practices. Scab prevalence was determined by scouting for symptoms, with a total of 84 farms surveyed in 2021. The incidence of bean scab was confirmed in all surveyed clusters, indicating its widespread occurrence across various agro‐ecological zones. Farmers exhibited common practices such as preference for uniform bean seeds (61%), use of uncertified seeds (83%), intercropping (80%), and limited crop rotation. Challenges included disease and pest infestations, with limited diversity in management options. Confirmation of the presence of bean scab in diverse agro‐ecological zones emphasizes its importance and the need for further research on its impact and epidemiology. Challenges with crop rotation were evident due to small farm sizes and subsistence‐focused farming. The study recommends further research for a comprehensive understanding of the link between increased scab importance and current bean farming practices such as short rotation periods and the use of susceptible varieties. Training programs are also vital to improve farmers' knowledge on safe agro‐chemical use, ensuring sustainable constraint management in common bean cultivation in Kenya.

## 1 SOCIETAL IMPACT STATEMENT

This study is the first look at the prevalence of scab caused by *Elsinoë phaseoli* in Kenyan bean fields and the accompanying bean cultivation practices, revealing the extensive presence of bean scab across various agro‐ecological regions. It emphasizes the pressing need for stakeholders and researchers to address this increasingly important disease. Furthermore, it brings attention to the obstacles encountered by smallholder farmers in implementing efficient crop management techniques. The study sets the groundwork for future inquiries into sustainable and tailored bean scab management, safeguarding the durability and output of common bean cultivation in Kenya.

## 2 INTRODUCTION

**FIGURE 1:**
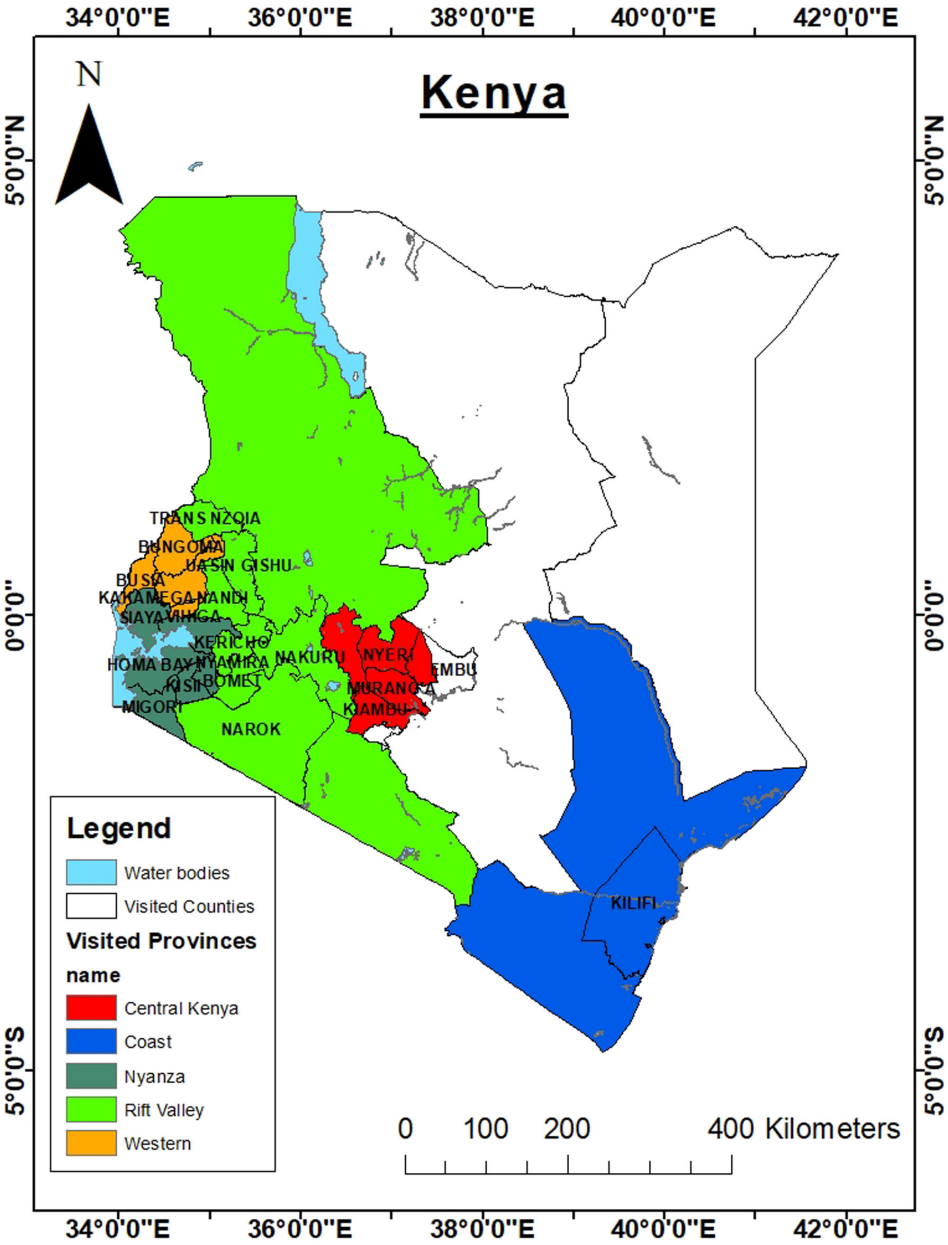
Major regions for dry bean production in Kenya.

Kenya holds the distinction of being the seventh‐largest global producer of common bean, and within East Africa, it ranks second (KenInvest, 2016). Common bean holds a crucial position as the second most important food crop in the country, trailing only maize. The cultivation of common bean is predominantly carried out by approximately 1.5 million smallholder farmers, collectively tending to nearly 1 million hectares of land, with an average yield of around 0.6 metric tons per hectare. Notably, key regions for dry bean production in Kenya include the former Rift Valley, Eastern, Nyanza, Western, and Central provinces (Figure 1), contributing 33%, 24%, 18%, 13%, and 20% of the national output, respectively (KenInvest, 2016).

Bean production in Kenya has been progressively increasing, albeit at a slower rate compared to Uganda and Tanzania. However, over the period from 2020 to 2022, there was a noticeable decline in the production metrics for dry beans in Kenya (FAOSTAT, 2023). The area harvested decreased from 1,147,709 hectares in 2020 to 1,042,288 hectares in 2022. Correspondingly, the yield per 100 g per hectare dropped from 6747 in 2020 to 4917 in 2022. This reduction in both areas harvested and yield resulted in a significant decline in overall production, from 774,365.67 tonnes in 2020 to 513,000 tonnes in 2022 (FAOSTAT, 2023). For smallholder subsistence farmers, the primary motivation behind common bean cultivation is to fulfill their dietary needs (Broughton et al., 2003). These farmers rely on common bean as their primary source of dietary protein, making it a staple crop grown in every cropping season.

The size of land cultivated by a household is reported to play a pivotal role in influencing the adoption of farming practices, as there exists a critical limit on farm size that affects smallholder farmers' preferences for specific farming technologies (Amare & Simane, 2017; Daberkow & McBride, 2003). Large farm sizes provide the flexibility to adopt various farming practices without running short of land, while smallholder farmers consistently rely on the same area of production across consecutive seasons (Atube et al., 2021). These limitations, coupled with poor farm management practices, compound various challenges such as soil fertility depletion, vulnerability to drought, weed competition, and the proliferation of pests and diseases, leading to what is termed as “legume yield depression syndrome” (Bainard et al., 2017; Juroszek & von Tiedemann, 2011).

In Kenya, common bean consumption is highest in the counties of Western Province (Kakamega, Bungoma, Busia, and Vihiga), where per capita consumption reaches 66 kg per year, compared to the national average of 14 kg per year. This reflects the region's greater reliance on beans as a dietary staple rather than as a supplementary or cash crop (Duku et al., 2020). According to the 2019 census, these counties have a population density of 648.4 people per km^2^, significantly higher than the national average of 82.0 people per km^2^ (Kenya National Bureau of Statistics, 2024). This high population density places considerable pressure on land resources, often leading to over‐cultivation without sufficient fallow periods. The resulting depletion of soil fertility and buildup of pathogen inoculum contributes to increased disease prevalence in the region (Gichangi et al., 2012; Ochichi et al., 2018).

Among these diseases, bean scab, caused by *Elsinoë phaseoli*, is particularly devastating, with Masheti et al. (2024a) first reporting its presence in the country. Yield losses due to bean scab can range from 70% to 100%, making it a major threat to Kenyan farms (Schwartz, 1991). *Elsinoë phaseoli* causes characteristic symptoms that are easily recognized by cork‐like necrotic tissues, which are often raised and display cracks, leading to the term “scabs” (Fan et al., 2017; Masheti et al., 2024b). On common beans, these scab‐like symptoms appear on all above‐ground parts of the plant and serve as a major diagnostic feature of the disease. In fact, leading mycologists in the mid‐20th century identified new species of *Elsinoë* solely based on these disease symptoms, without observing sporulation on the specimens (Jenkins et al., 1946). Notably, pod lesions are the most conspicuous symptoms (Masheti et al., 2024b).

Despite its considerable impact, the epidemiology and prevalence of bean scab, thought to be primarily endemic to Africa (Phillips, 1994), remain under‐researched, resulting in a limited knowledge base to address this challenge. The disease's prevalence in Kenya is largely undocumented, though unofficial reports from 1979 suggest it is widespread in bean‐growing regions (Mutitu, 1979). Given the importance of these areas in bean production, it is surprising that the disease has received minimal attention. This study aims to investigate the presence of bean scab caused by *E. phaseoli* in various bean‐growing regions of Kenya. Additionally, it seeks to document the challenges associated with bean cultivation on small farms, particularly focusing on the relationship between scab occurrence and current bean farming practices, including awareness of pest and disease issues and adaptation strategies.

## 3 MATERIALS AND METHODS

### 3.1 Study area

The survey sites encompassed major bean‐growing areas across five former provinces in Kenya: Western, Nyanza, Rift Valley, Central, and Coast. These areas spanned a variety of agro‐ecological zones (AEZs), illustrating the diversity of Kenya's agricultural landscapes. The zones ranged from the cooler, high‐altitude Upper Highland (UH) Zone 2 in Molo, Nakuru County (Rift Valley), to the warmer, more humid Coastal Lowland Zone 3–4 in Kilifi County (Coast). The classification of these zones follows the agro‐ecological zonation concept as defined by the FAO (1978), which considers factors such as altitude, mean annual rainfall, temperature, and evapotranspiration (Jaetzold et al., 2005).

In the Western region, surveys were conducted in Kakamega (Shinyalu), Vihiga, Bungoma (Saboti and Kibabii), and Busia (Nambale). In Nyanza, the surveyed areas included Kisii and Nyamira (Keumbu‐Keroka and Borabu), Homa Bay (Rongo), Siaya, and the wider Ndiwa‐Migori‐Suba‐Kuria region. In Rift Valley, surveys were carried out in Uasin Gishu (Moiben), Nakuru (Njoro, Bahati to Subukia, Lanet, and Rongai), Trans‐Nzoia (Kiminini), and Narok (Ndabibi). In Central Kenya, the study covered Muranga (Maragua), Embu (including Kiritiri), Kiambu (Limuru, Mwangu, Gatundu, Githunguri, and Kimuvu), and Nyeri (Karatina). The coastal region was represented by Kilifi (Fundi‐Isa).

The study areas were then categorized into two groups based on perceived dependence on common beans, taking into account both consumption rates and population density. Western Kenya (Kakamega, Bungoma, Busia, and Vihiga counties) was selected for its high common bean consumption (66 kg per capita per year) (Duku et al., 2020) and a population density of 648.4 people per km^2^ (Kenya National Bureau of Statistics, 2024). The remaining regions of the country, with an average population density of approximately 360.0 people per km^2^ (Kenya National Bureau of Statistics, 2024), were grouped into a second category for comparison.

### 3.2 Survey procedure

The study targeted farmers in January 2022 and February 2023, after the 2021 bean scab prevalence survey, which focused on the late stages of the long‐rains (May–June) and short‐rains (October–December) bean growing seasons. The survey employed a three‐stage sampling design. Major common bean‐growing regions were purposively selected to form the primary sampling units (PSUs) organized into four clusters: the Western cluster, the Rift Valley cluster (comprising Trans‐Nzoia, Uasin Gishu, Nandi, Bomet, Kericho, Narok, and Nakuru counties), the Nyanza cluster (encompassing Siaya, Migori, Homa Bay, Kisii, and Nyamira counties), and the Central cluster (made up of Nyandarua, Laikipia, Nyeri, Murang'a, Embu, and Kiambu counties). Rift Valley, Nyanza, and Central clusters represented other areas. Agro‐ecological zones (AEZs) for bean cultivation were deliberately selected from each primary cluster to constitute the secondary sampling units (SSUs).

At the third level of sampling, the ultimate sampling units (USUs) for the scab prevalence survey were identified by scouting common bean farms with bean plants at the podding stage. These farms were situated at least 10 km apart. For the respondents' survey, bean farmers who had cultivated common bean in the immediate previous season were considered as USUs. Since the precise population size of farms and farmers meeting the study criteria was unknown, sample sizes were determined using Roscoe's rules of thumb for sample size determination (Roscoe, 1975), as proposed by Memon et al. (2020). A total of 128 bean farmers were surveyed, with 61 farmers selected from the Western cluster and the remaining 67 farmers distributed among the other clusters, comprising 24 for the Rift Valley cluster, 22 for the Nyanza cluster, and 21 for the Central cluster. For the bean scab prevalence study, the sample size was 84 farms distributed across the four cluster regions as follows: 21 farms in the Western cluster in Agro‐ecological zones Lower Highland (LH) 3, Upper Midland (UM) 1, UM 2, UM 4, Lower Midland (LM 1), and LM; 18 farms in the Nyanza cluster in Agro‐ecological zones LH 1, LH 2, UM 1, LM 1, LM 3, and LM 4; 21 farms in the Rift Valley cluster in Agro‐ecological zones UH 2, LH 1, LH 2, LH 3, LH 4, UM 2–4, and UM 4; and 24 farms in the Central cluster in Agro‐ecological zones LH 1, LH 2, UM 1–2, UM 2, UM 3, UM 3–4, and LM 4, as well as coastal lowland zone 3–4 in Kilifi county.

**FIGURE 2:**
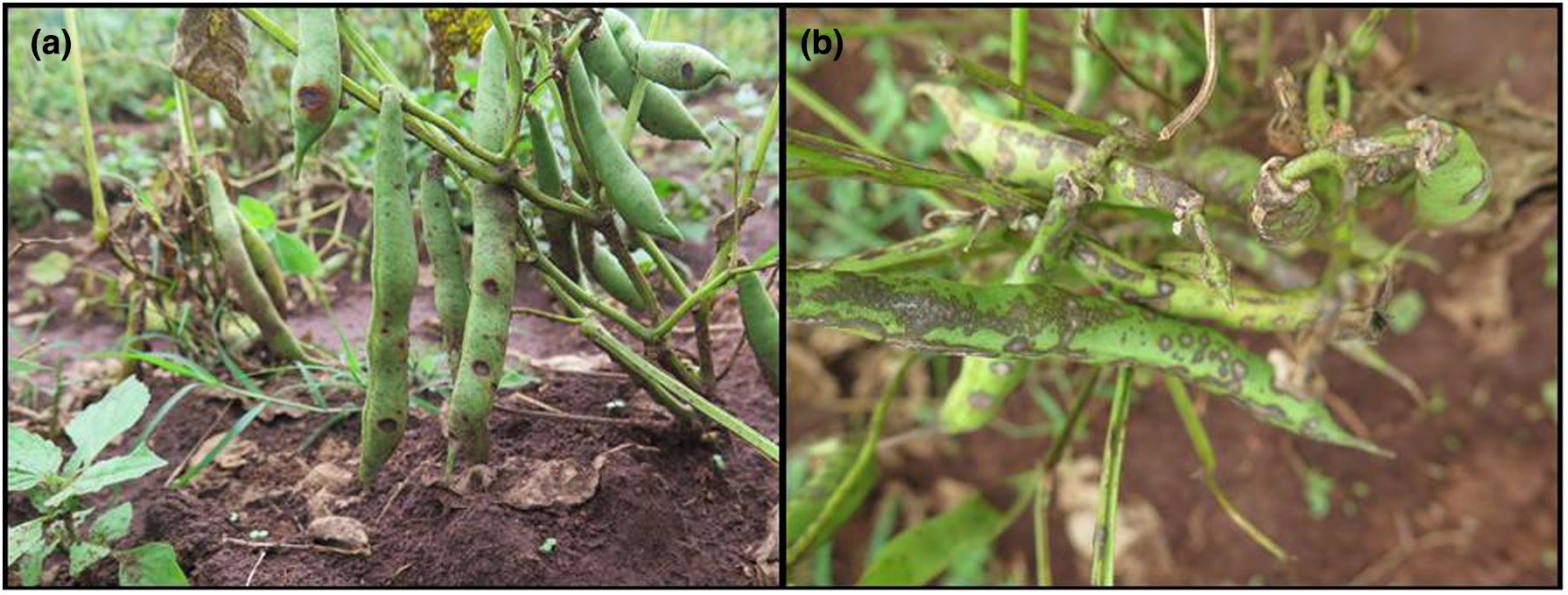
Common bean pods infected by (a) bean anthracnose caused by *Colletotrichum lindemuthianum* (b) bean scab caused by *E. phaseoli*.

Agricultural extension officers familiar with the study areas identified farmers for contact based on their fit for Ultimate Sampling Units criteria of having cultivated beans in the previous season. A semi‐structured questionnaire, pre‐tested on an earlier cohort of farmers for optimization, was administered to the respondents through a combination of face‐to‐face and phone interviews. The questionnaire gathered information on various aspects, including respondents' gender, farm size, area under common bean cultivation, seed sources, cropping systems, awareness and perception of bean farming challenges, and awareness and application of pest and disease management strategies. Additionally, colored photographs displaying bean scab and bean anthracnose (*Colletotrichum lindemuthianum*) symptoms on leaves, stems, and pods were shown to the respondents to assess their ability to differentiate between the two fungal diseases (Figure 2).

The consent process for survey participants was conducted verbally. Prior to commencing the survey, each participant was provided with a verbal explanation of the study's objectives in their primary language, procedures, and potential benefits. Participants were informed that their participation was voluntary and that they had the right to withdraw at any time without penalty. They were also assured that their responses would be kept confidential and anonymized. During the verbal consent process, participants were encouraged to ask questions, and any inquiries raised were addressed to ensure a clear understanding of the study. Upon obtaining verbal consent from each participant, the survey was then administered. The verbal consent obtained from each participant was documented by recording their affirmative response or acknowledgment. This documentation included noting the participant's agreement to participate, as well as the date of consent. To ensure consistency and accuracy in the consent process, the researcher followed a standardized script, which was designed to provide comprehensive information about the study while allowing for flexibility to address any participant queries or concerns.

Disease prevalence was determined by scouting for common bean plants exhibiting characteristic bean scab symptoms, with particular emphasis on pod symptoms, which were the most easily identifiable. The farms were traversed in zigzag directions, covering at least 20 meters; in many cases, larger distances were covered for thoroughness. Fields were marked present when bean scab symptoms were observed and absent when no scab symptoms were recorded. A non‐random sampling method was employed, with only pods displaying scab symptoms selected from each farm and transported to the laboratory for further examination to confirm the presence of *Elsinoë phaseoli*. GPS data points were obtained for each sampling location.

### 3.3 Data analysis

Data captured in the questionnaires was analyzed using the R software (R Core Team, 2022). A chi‐squared test was conducted to compare the responses between the western cluster and the rest of the country (other clusters). Mean comparisons were conducted at a 95% confidence level. Bean samples taken to the laboratory were closely examined under both light‐ and stereo microscopes.

## 4 RESULTS

### 4.1 Prevalence of bean scab in Kenya

The bean scab symptoms were recorded in all clusters and counties, with the exception of Nyeri, Nyandarua, and Embu in the Central cluster, which were off‐timed (OT) due to delayed rains and therefore had young crops during the survey, making pods unavailable for inspection (Table 1). Scab occurred in all agro‐ecological zones (AEZs) between LH 1 and LM 4. Among the major bean‐growing AEZs, only UH 2 (Molo‐Elbergon) did not show scab symptoms in the selected farms (Table 1). Despite the presence of scab in the fields, the surveyed respondents could not convincingly differentiate between scab and anthracnose and this part of the survey was discontinued.

**TABLE 1:** Regions/Clusters and Agro‐ecological zones surveyed for the prevalence of bean scab in Kenya.

Clusters	AEZs	County (area name)	Present	Absent
Western	UM 1	Kakamega (Shinyalu), Nandi (Bonjoge), Vihiga	*	
	UM 2	Bungoma (Saboti)	*	
	LM 1	Busia (Nambale)	*	
	LM 2	Bungoma (Kibabii)	*	
	LH 1	Kisii‐Nyamira (Keumbu‐Keroka)	*	
	LH 2	Kisii‐Nyamira (Borabu)	*	
Nyanza	UM 1	Kisii (Keumbu)	*	
	LM 1	Homa bay (Rongo)		*
	LM 3	Ndiwa‐Migori‐Suba‐Kuria	*	
	LM 4	Siaya	*	
	UH 2	Molo UH 2 (Molo, Elbergon)		*
	LH 1	Kericho‐Bomet	*	
	LH 2	Nakuru (Bahati to Subukia)	*	
Rift valley	LH 3	Uasin Gishu, Nakuru (Njoro)	*	
	LH 4	Narok‐Nakuru (Ndabibi)	*	
	UM 4	Trans‐Nzoia (Kiminini), Nakuru (Lanet, Rongai)	*	
	LH 1	Kiambu (Limuru, Mwangu)	OT	
	UM 1–2	Kiambu (Gatundu, Githunguri, Kimuvu)		*
	UM 2	Nyeri (Karatina)	OT	
Central	UM 3	Muranga UM 3 (Maragua)	*	
	UM 3–4	Embu	OT	
	LM 4	Embu (Kiritiri)	OT	
Coastal	Semi‐humid to semi‐arid	Kilifi (Fundi‐Isa)	*	

Abbreviations: AEZs, agro‐ecological zones; LH, lower highlands; LM, lower midland; OT, off‐timed; UH, upper highlands; UM, upper midland. *Indicates the presence or absence of bean scab as confirmed during the survey in the respective clusters and agro‐ecological zones.

**FIGURE 3:**
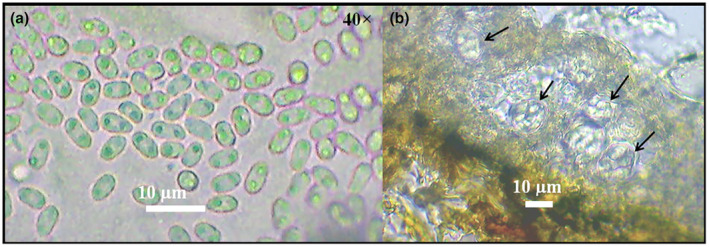
(a) *Elsinoë phaseoli* single‐celled, oblong‐elliptical and mostly biguttulate conidia. (b) Globulose to sub‐globulose bitunicate asci, irregularly arranged in locules of order Myriangiales.

Disease was markedly present only when characteristics of cork‐like necrotic scab tissue were observed on pod surfaces and samples collected. Thin cross‐sections of the collected infected plant tissue examined under a microscope revealed raised ascomata primarily on the epidermal layer. These ascomata displayed a range of colors from gray to brown to red, sometimes even showing mixed coloration. Within these ascomata, numerous locules were present. Inside these locules, bitunicate asci with a globulose to sub‐globulose shape, measuring up to 30 μm in diameter, were irregularly arranged in single or multiple layers. The ascospores, which had dimensions of up to 10 × 5 μm, varied in color from hyaline to brownish. They were trans‐septate (having 1–3 septa) or muriform and were positioned irregularly within the asci. The ascospores were only released when the stromatal layers above them disintegrated. Additionally, pseudoparenchymatous acervuli were observed on the surface of the parasitic ascomata. These acervuli contained hyaline to pale‐brown conidiophores. These conidiophores produced hyaline, single‐celled, oblong‐elliptical conidia, which were typically biguttulate and had measurements of up to 6 × 2 μm (Figure 3).

### 4.2 Characteristics and farming habits of surveyed respondents

The study found that gender and region were independent factors, with no significant difference (*p* < .05) between the number of males and females in each region. In Western Kenya, the female‐to‐male ratio was 1.18, while in other regions, it was 1.31. Additionally, there was no significant difference (*p* < .05) in the sizes of areas under bean cultivation or bean yields in each region, with the average area of land dedicated to bean cultivation being 0.25 ha. Various aspects of farming habits, including variety composition, seed type, seed source, cropping composition, and crop rotation, were consistent across the regions, as evidenced by the lack of significant differences (*p* < .05) observed between Western and other clusters (Table 2).

**TABLE 2:** Comparisons of farming habits of respondents (farmers surveyed; *n* = 128) in Western Kenya and the rest of the country.

Responses		Western Kenya (*n* = 61)	Other areas (Rest of the country) (*n* = 67)	Mean (*n* = 128)	Chi‐square *χ* ^2^ (*p*‐value)
Variety composition	Mixed	39	39	39	0.006 (.937)^ns^
	Uniform	61	61	61	
Seed type	Certified	12	22	17	2.426 (.119)^ns^
	Uncertified	89	78	83	
Seed source	From Other farmers	15	3	9	7.082 (.132)^ns^
	Grain Market	33	43	38	
	NGO	2	3	2	
	On‐farm Saved	41	37	39	
	Certified seed seller	10	15	12	
Cropping composition	Intercrop	85	75	80	2.224 (.136)^ns^
	Pure stand	15	25	20	
Crop rotation	Rotates	54	56	55	0.059 (.809)^ns^
	No rotation	46	44	45	
Rotation period	6–12 months	10	34	22	10.737 (.005)**
	3–6 months	38	21	30	
	Seasonal	52	44	48	

Abbreviations: *n*, sample size; ns, not significant at *p* ≤ .05.

** Significant at *p* ≤ .05.

**FIGURE 4:**
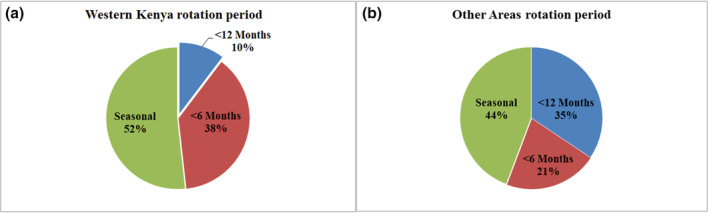
Subsistence bean farmers' rotation periods in (a) Western Kenya and (b) other regions of Kenya.

The majority of respondents cultivated uniform varieties (61%) compared to those who cultivated mixed varieties (39%). Moreover, 83% of respondents used uncertified seed, with only 17% utilizing certified seed. The primary sources of seeds were on‐farm‐saved seeds (39%), followed by seeds from grain markets (38%). Other seed sources included seeds obtained from other farmers, non‐governmental organizations, and certified seed sellers, accounting for 9%, 2%, and 12%, respectively. As for cropping composition, 80% of the total respondents practiced intercropping, while 20% cultivated beans in pure stands. The overall ratio of farmers who practiced crop rotation to those who did not was 1.23, with 86% of the latter citing a lack of space as the reason for not using the practice. Rotation periods varied by region, with significant differences (*p* < .05) observed between the rotation periods in each region. A seasonal rotation period was the most popular in both Western Kenya and other areas, with 52% and 44%, respectively. This was followed in popularity by rotation periods of three to 6 months and 6–12 months, at 38% and 10% in Western Kenya and 21% and 34% in other areas, respectively. The maximum reported rotation period was 12 months (Figure 4).

### 4.3 Respondents' awareness and perception of bean farming challenges

The perception of challenges faced by farmers was consistent across the regions (Table 3). Overall, 88% of respondents reported facing various challenges in common bean cultivation, while 12% stated that they faced no challenges. The nature of these challenges differed among regions, with 35% of those who faced challenges in Western Kenya mentioning “Barafu” as the main challenge, followed by “leaf curl” (19%) and “leaves turning yellow” (7%). Other challenges mentioned by 19% of respondents in Western Kenya included “hailstones,” “insufficient rainfall,” “excessive rainfall,” “red‐black coloration,” and “stunted growth.” In other areas, 49% of those who faced challenges identified “pests” as the main issue, followed by ‘leaves turning yellow’ (26%), ‘Barafu’ (20%), and “leaf curling” (6%). The farmers' perception of the impact of these challenges on yield was independent of the regions, with no significant difference (*p* < .05) between them in each region. Overall, 87% of farmers claimed that their yields were negatively impacted, while 13% reported no negative impacts.

**TABLE 3:** Comparison of pests and diseases management awareness and application by respondents (farmers surveyed; *n* = 128) in Western Kenya and other areas.

Responses		Western Kenya (*n* = 61)	Other areas (*n* = 67)	Mean (*n* = 128)	Chi‐square *χ* ^2^ (*p*‐value)
Challenge perception	Faces challenges	85	90	88	0.740 (.390)^ns^
	No challenges faced	15	10	12	
Challenge description	“Barafu”	35	20	27	19.853 (.0005)**
	Leaf curling	19	6	12	
	Leaf yellowing	7	26	16	
	Insect pests	21	49	35	
	Other challenges	19	0	9	
Yield impact perception	Yield negatively impacted	91	84	87	0.786 (.375)^ns^
	Yield not negatively impacted	9	16	13	

Abbreviations: *n*, sample size; ns, not significant at *p* ≤ .05.

** Significant at *p* ≤ .05.

The awareness and application of pest and disease management strategies by farmers were consistent across the regions, as evidenced by the lack of significant differences observed between the two regions (*p* < .05). Overall, 58% of farmers reported being aware of at least one management option for the challenges they faced, compared to 42% who had no knowledge of available management options. Among the respondents who were aware of available management options, 90% used commercial chemical options, while 10% used non‐chemical alternatives. Only 5% of the farmers applied chemicals at the product label recommended interval, with the majority (48%) opting to apply chemicals only when symptoms were present. Other popular application intervals were every 14 or bimonthly (29%) and every 7 days or weekly (19%).

## 5 DISCUSSION

Unmistakable bean scab symptoms were observed in all the surveyed bean‐growing clusters, ranging from agro‐ecological zone LH 1 to LM 4 and the semi‐humid area of Kilifi. A thorough examination of the collected scab lesions from each location revealed both sexual and asexual pathogen features consistent with the Myriangiales order within the class Dothidiomycetes, to which the genus *Elsinoë* belongs (Braga et al., 2020; Fan et al., 2017). The Myriangiales order is distinguished by pulvinate, irregular ascostromata with compartments containing irregularly arranged asci in single or multiple layers, each containing one or more asci. The asci are characterized by minute pedicels and indistinct ocular chambers, while the irregularly arranged ascospores are released only upon the rupture of the stromatal layers above them (Hyde et al., 2013; Li et al., 2011). Additionally, hyaline, single‐celled, oblong‐elliptical conidia, primarily biguttulate, were also observed. These descriptions correspond with those provided by Phillips (1994) and Masheti et al. (2024b) for *Elsinoë phaseoli*.

It is crucial to highlight that this assessment was based solely on visual symptoms and lacks subsequent verification through molecular analyses or inoculations for samples from each region. However, confidence is placed in the observed symptoms, as *Elsinoë* species are known to cause “signature‐bearing diseases” that are easily recognizable by their symptom‐markers, specifically cork‐like necrotic tissues referred to as scabs (Fan et al., 2017). Bean scab symptoms, which appear on all above‐ground plant parts, are most conspicuous on pods, the focus of this study, and serve as a reliable means of disease recognition (Allen et al., 1996; Masheti et al., 2024b; Phillips, 1994). Furthermore, in the mid‐20th century, leading mycologists working with *Elsinoë* successfully identified new species based solely on disease symptoms, without observing sporulation on the specimens (Jenkins et al., 1946). This study is part of the work by Masheti et al. (2024a) that first reported the occurrence of bean scab caused by *E. phaseoli* in Kenya. In that study, scab symptoms were conclusively linked to *E. phaseoli* through successful phenotypic and genotypic characterization of the pathogen from samples obtained from agro‐ecological zone UM 2 of Kakamega County in the Western cluster. Koch's postulate was also fulfilled for this isolate, validating the use of symptomatology for the rest of the regions.

A study by Mwangombe et al. (2007) covering 13 different agro‐ecological zones (from LH1 to LM5) in five districts found angular leaf spot (*Phaeoisariopsis griseola*) on beans in all locations, demonstrating how a fungal disease can prevail across diverse environments where the host is cultivated, irrespective of perceived differences in environmental conditions. Like *P*. *griseola*, *E. phaseoli* may also exhibit similar variability in adaptability. A study by Mchau et al. (1998) on *E. phaseoli* in South Africa revealed geographical groupings among isolates, indicating potential adaptability variations. Further research is needed to fully understand the adaptability of *E. phaseoli*.

Among the surveyed agro‐ecological zones (AEZs), only UH 2, which encompasses Molo and Elbergon, did not report scab incidences. The area of Molo (UH 2) with an average yearly temperature of 18.6°C, which is 4% lower than Kenya's overall average temperature, has a higher altitude and lower average temperatures than the other regions studied. The development of a disease is contingent upon favorable environmental conditions, including temperature, humidity, and sunshine hours (Ahmed et al., 2016; Bharti et al., 2019; Eshetu et al., 2018). It is important to note that members of the *Elsinoë* genus are known to be temperature‐sensitive. For instance, Li et al. (2018) observed that temperature is critical for conidial formation in *E. ampelina*, with conidial production occurring at temperatures ranging from 2 to 40°C, with optimal sporulation at 20°C (Carisse & Lefebvre, 2011). While these observations might hint at a temperature threshold for bean scab occurrence, it remains inconclusive, and more research on the influence of temperature and other environmental factors on *E. phaseoli* is needed.

The reported farming practices and experiences of respondents across the regions revealed several commonalities in terms of variety composition, seed type and source, cropping composition, and crop rotation. The majority of farmers (61%) preferred uniformity in their bean varieties, with 75% cultivating uncertified seeds sourced from their own farms or local grain markets. It is essential to clarify that “uniform variety” in this context does not imply a single genotype. Instead, the designation of ‘uniform variety’ versus “mixed variety” was a subjective categorization made by the farmers, who tend to group genotypes with similar physical traits together. This makes it unreliable to ascertain genetic uniformity, especially when seeds are primarily obtained from informal sources, as was the case in this study. Previous research has found that the most popular attributes of common beans demanded by farmers are drought and disease tolerance, yield, taste, and cooking time (Katungi et al., 2015).

The high preference for uncertified seeds aligns with findings from other studies (Wilkus et al., 2017, 2018). Muthoni and Nyamongo (2008) reported that approximately 78% of all seeds used in Kenya originate from the informal sector. Other sources of seeds identified in this study included other farmers and non‐governmental organizations, consistent with research on farmers' seed acquisition in Kenya (Tripp, 2000). The preference for informal seed sources could be attributed to the relatively high cost of seeds from formal sources or the desirable traits of local beans that are well adapted to local climatic conditions. Farmers engaged in commercial bean production tend to opt for formal seed sources for their potential benefits, while those focused on subsistence farming rely on seeds saved from their own production or informal sources (Muthoni & Nyamongo, 2008). Notably, research by Nassary et al. (2020a) demonstrated that seeds obtained from local informal sources can yield significantly more than certified bean seeds, possibly due to the adaptability and stress tolerance mechanisms of local bean varieties (Nassary et al., 2020b).

Nearly 80% of the farmers reported growing beans in intercropped systems with other crops. Intercropping allows farmers to make the most of their available land by meeting diverse nutritional and economic needs (Pretty et al., 2011). For instance, intercropping maize with beans enables farmers to meet both their carbohydrate and protein dietary needs, while intercropping coffee with beans allows them to address dietary and commercial or economic requirements. Intercropping beans with cereals has been effective in controlling diseases in beans, as reported by Olango et al. (2016), Eshetu et al. (2018), and Mengesha and Yetayew (2018). Boudreau (2013) highlighted that the effects of intercropping mimic the heterogeneity of plant communities, impacting disease dynamics by altering host density, wind speed, vector spread, and microclimate factors like temperature, relative humidity, and leaf wetness. Common bean can also benefit secondary crops in intercropped systems through nitrogen fixation, improved soil properties, increased mycorrhizal infection, protection against diseases and pests, and reduced costs associated with purchasing nitrogen‐containing fertilizers (Franke et al., 2016; Gan et al., 2015; Ojiem et al., 2014; Rurangwa et al., 2018).

Surprisingly, despite 55% of farmers reporting the practice of crop rotation, none reported a rotation period exceeding 1 year. Crop rotation is widely regarded as the most effective cultural practice for controlling bean diseases and is often recommended (Hall & Nasser, 1996; Juroszek & von Tiedemann, 2011). It is advised that beans should not be planted in the same field more than once every 3 years to prevent disease recurrence (Palti, 1981). This practice has been found to be moderately to highly effective in managing 33 out of 50 bean diseases (Palti, 1981). In this study, farmers preferred a seasonal rotation aligned with the rainy season, typically lasting less than 6 months (Jaetzold et al., 2005). However, Masheti et al. (2024c) found that this 6‐month rotation period was inadequate for controlling bean scab. In contrast, fields that had rotated with non‐legume crops for 3 years showed complete suppression of the disease. As a result, none of the respondents in this study practiced an adequately long rotation. *Elsinoë* spp. can overwinter in crop residues, serving as the primary source of inoculum for new infections when both susceptible hosts and favorable environmental conditions are present (Carisse & Provost, 2024). Additionally, without proper crop rotation, other cultural and chemical management strategies may prove ineffective in controlling bean scab (Masheti et al., 2024c).

Furthermore, 86% of those who did not practice crop rotation cited space limitations as the primary reason. With an average farm size of 0.25 hectares, most respondents fall into the category of smallholder subsistence farmers, as defined by the Food and Agriculture Organization (2013). For these farmers, the primary reason for cultivating common beans is to meet their dietary needs (Broughton et al., 2003). This implies that for most farmers, going an entire season without cultivating beans, which serve as their primary source of dietary protein, is improbable. This practical constraint makes longer rotation periods unfeasible. Research by Amare and Simane (2017) in the Muger River sub‐basin of Ethiopia indicates that the size of land cultivated by a household tends to influence the adoption of farming practices. Additionally, Daberkow and McBride (2003) showed that there is a critical limit on farm size for smallholder farmers, affecting their ability to adopt certain farming technologies. Consequently, continuously growing legumes in the same field or practicing insufficient crop rotation can lead to the buildup of various pests and diseases and increase disease severity (Bainard et al., 2017; Juroszek & von Tiedemann, 2011; Masheti et al., 2024c).

Respondents shared similar experiences in terms of the challenges they faced in bean farming and their perceived impacts on yields. Overall, 88% of farmers reported facing challenges during bean cultivation, with 87% of this group indicating that the challenges they encountered had a negative impact on their bean yields. Common bean productivity in Kenya faces challenges from a range of biophysical stresses, including climatic unpredictability, soil fertility loss, and pest and disease pressures (Katungi et al., 2011). Although the descriptions of the challenges faced by farmers differed by region, as the names of pests and diseases are often regional, “Barafu” (27%) and “insect pests” (35%) were among the most frequently reported challenges. In Western Kenya, “leaf curling” was reported by 18.6% of the respondents, while in other areas, “leaves yellowing” was commonly reported by 26% of the respondents.

It was discovered during the preliminary survey for this study that farmers often used the term ‘Barafu’ (cold) to describe diseases occurring during periods of exceptional wetness. In several cases, these diseases turned out to be bean anthracnose (*C. lindemuthianum*), CBB (*Xanthomonas axanopodis* pv. *phaseoli*), and bean scab. “Leaf curling” also encompassed symptoms of both bean common mosaic Potyvirus and Bean scab (*E. phaseoli*). Furthermore, many farmers struggled to differentiate between various symptoms. Other diseases known to occur in at least one of the surveyed regions include: Rust (*Uromyces appendiculatus*), floury leaf spot (*Mycovellosiella phaseoli*), *Cercospora* leaf spots, halo blight (*Pseudomonas syringae* pv. *phaseolicola*) and bacterial brown spot (*Pseudomonas syringae* pv. *syringae*) (Allen et al., 1996).

A wide range of pests cause damage to bean fields in Kenya, including *Aphis* spp., the legume pod borer (*Maruca testulalis* or *Vitrata Fab*) bean fly (*Ophiomyia* spp.) flower bud thrips (*Megalurothrips sjostedti* Tryomb), a host of plant‐sucking bugs, (*Clavigralla tomentosicollis* Stal, *Anoplocnemis curvipes* Fab, *Aspavia armigera* Fab, *Nezera viridula*, *Riptortus dentipes*), leaf hoppers (*Empoasca dolichi* and *E. lybica*) and white flies (*Bemisia tabacci*) (Allen et al., 1996; Ogecha et al., 2019). The damage inflicted by insect pests feeding on legumes in East Africa is estimated to result in approximately 70% yield loss (Edema & Adipala, 1996). The significance of individual insect pests varies from one region to another and is influenced by environmental and agronomic conditions (Aslam et al., 2007; Songa & Ampofo, 1999).

The study also revealed that farmers' knowledge concerning the mitigation of constraints observed in common bean fields was limited and needed diversification. Approximately 58% of the respondents stated that they were aware of management options to address the challenges they faced. However, a substantial 90% of these respondents reported being aware of only commercial chemical mitigation options. Interestingly, less than 5% of the respondents who used commercial chemicals adhered to product label recommendations for their application. This high preference for chemical pesticides among Kenyan farmers is consistent with findings by Constantine et al. (2020), where 87% of farmers reported using them to manage various crop pests. The reasons behind this preference are attributed to perceptions of effectiveness, particularly in terms of speed of action and the spectrum of activity, as well as the availability and affordability of chemical pesticides.

It is worth noting that a lack of knowledge regarding proper chemical use among smallholder farmers is a widespread issue. For instance, a study in Eastern Kenya found that 94% of farmers chose chemical options to control grain legume pests (Abtew et al., 2016). Abtew et al. (2016) further found that 20% of the respondents did not even know the names of the chemicals they used to spray their grain legumes. This knowledge gap can be attributed to factors such as the language barrier and the technical complexity of chemical instructions. Therefore, there is a pressing need for awareness campaigns, training programs, and promotional efforts to enhance farmers' knowledge regarding the proper use of agrochemicals. Approaches and concepts such as integrated pest management, agro‐ecological crop protection, organic agriculture, and pesticide‐free (but non‐organic) production systems need to be developed and disseminated to address smallholder bean farmers' unique situations. These methods will reduce pesticide use and associated risks among farmers by ensuring effective pest management and maintaining high productivity (Finger et al., 2024). They also aim to minimize environmental impacts and human health risks while ensuring the economic viability of common bean production.

Bean scab is prevalent in all major bean‐growing regions in Kenya, posing significant challenges to bean farmers across different environments. Research is needed to understand its variability and impact. Common farming practices among small‐scale bean growers might increase susceptibility to scab. Farmers face various challenges, including limited availability and knowledge of management options, and an over‐reliance on agro‐chemicals, often used improperly. The difficulties farmers encounter in practicing crop rotation due to small land sizes, along with the effects of inadequate crop rotation, may be the biggest risk factors for bean scab.

## CONFLICT OF INTEREST STATEMENT

The author(s) declare no conflict of interest.

## Data Availability

The datasets generated and analyzed in this study are available in the figshare repository: https://doi.org/10.6084/m9.figshare.27100093.
